# Case Report: [^18^F]PI2620 as a Tau Imaging Agent in Posterior Cortical Atrophy

**DOI:** 10.3389/fneur.2020.566667

**Published:** 2020-12-08

**Authors:** Yu Kong, Kexin Xie, Hongwen Qiao, Yue Cui, Donglai Jing, Yuting Wang, Xuying Li, Liyong Wu

**Affiliations:** ^1^Department of Neurology, Xuanwu Hospital, Capital Medical University, Beijing, China; ^2^Department of Nuclear Medicine, Xuanwu Hospital, Capital Medical University, Beijing, China

**Keywords:** posterior cortical atrophy (PCA), MAPT mutation, Tau PET, [^18^F]PI2620, dementia

## Abstract

Posterior cortical atrophy (PCA) is widely considered as an atypical variant of Alzheimer disease and is characterized by a progressive decline in visual function. PCA has been investigated from the standpoints of brain structure and metabolism, but tau deposition and its relationship to disease severity still remain unclear. Here, we used a novel tau ligand, [^18^F]PI2620, to visualize tau deposition in a PCA patient. The results showed that high [^18^F]PI2620 uptake in posterior cortical regions was associated with clinical manifestations, morphologic changes in the brain observed by magnetic resonance imaging (MRI), and hypometabolism detected by [^18^F] fluorodeoxyglucose (FDG) positron emission tomography (PET). This is the first report demonstrating a clinical anatomical correspondence between [^18^F]PI2620 PET results, clinical manifestations, MRI, and [^18^F]FDG PET findings in a Chinese patient with PCA. The results also support the utility of [^18^F]PI2620 for visualizing tau aggregation in PCA.

## Introduction

Posterior cortical atrophy (PCA) is a neurodegenerative syndrome affecting parietal, occipital, and occipitotemporal cortices that is characterized by visuospatial and visuoperceptual impairment, while episodic memory, judgment, and insight are relatively well-preserved early in the clinical course (1).

As an atypical Alzheimer disease (AD), PCA includes the amyloid plaques and neurofibrillary tangles seen in typical amnestic AD. Recent studies have reported a close relationship between tau neurofibrillary tangles and clinical deficits at autopsy, suggesting that tau pathology induces subsequent neurodegeneration. The development of selective tau tracers has made it possible to qualitatively analyze brain tau profiles *in vivo* (2–4). [^18^F]PI2620 is a next-generation tau-specific positron emission tomography (PET) tracer with high binding affinity for pathogenic tau protein but without off-target binding to β-amyloid, monoamine oxidase (MAO)-A, or MAO-B (5). Human studies have shown that [^18^F]PI2620 can detect tau pathology throughout the course of AD (6, 7). However, there have been few studies using [^18^F]PI2620 PET in PCA, and its utility in this context has yet to be demonstrated. Here, we described the case of a Chinese PCA patient presenting with typical visual disorder and described his magnetic resonance imaging (MRI), [^18^F] fluorodeoxyglucose (FDG) PET, [^18^F]AV45 PET, and [^18^F]PI2620 PET outcomes and neuropsychologic test results. We hypothesized that higher [^18^F]PI2620 uptake is related to clinical manifestation, atrophy patterns observed by MRI, and hypometabolism detected by [^18^F]FDG PET.

## Case Report

A 56-year-old right-handed man with secondary education was admitted to our hospital complaining of visual deficit that had gradually progressed over 6 years. Initial presentation 6 years prior included misplacement of his belonging such as keys and wallet; however, as his daily life activities were not affected and he maintained independence, he continued driving and working full time. The symptoms were not sufficiently severe for the patient to seek treatment at that time. Four years ago, he started experiencing confusion at home, which led him to enter the wrong rooms; he urinated anywhere as he was unable to locate the bathroom. As the symptoms worsened, he could no longer find a cup on the table immediately in front of him and gradually developed difficulty in writing in a given area on a piece of paper. He could not track sentences in the newspaper and started listening to the radio instead of watching television. He subsequently experienced problems in performing calculations, dressing himself, and recognizing faces. He had to cease working and driving because of difficulties in identifying common objects. On the other hand, his ability to recognize different sounds and voices was preserved, and he did not experience any hallucinations, weakness, extrapyramidal symptoms, or ataxia. There were no significant events in his medical history. By probing his family history, it was revealed that his mother suffered from mild cognitive decline at 80 years old, while his father did not have any neurologic dysfunction.

Neurologic examination revealed no cranial nerve, motor, or cortical sensory impairment. The patient's performance in neuropsychologic tests (Table 1) showed visuospatial deficits, prosopagnosia, color agnosia, and simultanagnosia, memory deficits, dyscalculia, agraphia, and alexia. The patient had difficulty completing some tests such as the trail making, line bisection, and word reading tests, as he was unable to recognize the figures. Language and executive functions were less affected. The patient was aware of his visual deficits and became silent and withdrawn as the disease progressed.

**Table 1:** Neuropsychologic profile of the patient.

**Test**	**Patient's score/maximum score**
Mini-mental state examination score	10/30
Montreal cognitive assessment scale score	5/30
Boston naming test (30 items)	7/30
Clock drawing test	0/3
Trail making test A	Unfinished (non-cooperation)
Trail making test B	Unfinished
Rey complex figure test	0/16
Forward digit-span task	5/10
Backward digit-span task	3/8
Immediate memory	8/45
Delayed memory	0/15
Face identification	4/10
Color identification	4/10
Word reading test	Unfinished
Line bisection test	Unfinished
Clinical dementia rating (CDR)	2

**Figure 1:**
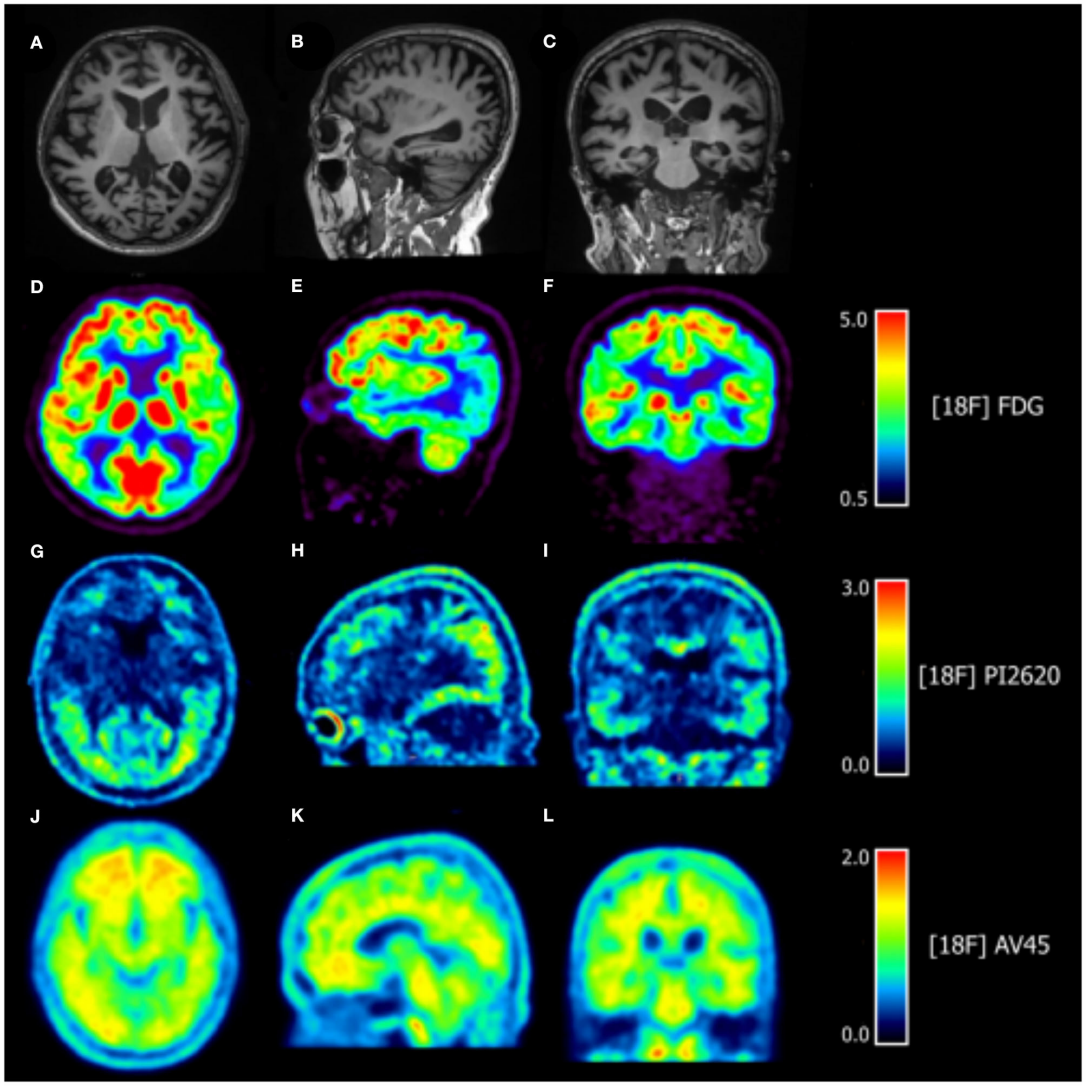
Axial, sagittal, and coronal slices from MRI and [^18^F] fluorodeoxyglucose ([^18^F]FDG), [^18^F]PI2620, and [^18^F]AV45 PET scans. **(A–C)** T1-weighted sequences in MRI show cortical and subcortical atrophy, especially in bilateral parietal and occipital lobes. **(D–F)** [^18^F]FDG PET revealed significant hypometabolism in diffuse regions of bilateral temporal, parietal, and occipital lobes, prominently in occipital lobes, and no metabolic change was observed in the frontal lobes, basal ganglia, and thalami. **(G–I)** [^18^F]PI2620 PET showed increased tracer uptake throughout the brain, especially in the temporal, parietal, and occipital lobes. **(J–L)** [^18^F]AV45 PET showed amyloid deposition throughout the cortex that was especially pronounced in bilateral frontal cortices.

Routine laboratory test showed that blood and urine parameters, serum chemistry, and liver and renal functions were within normal limits. Lumbar puncture revealed normal cerebrospinal fluid pressure and biochemistry. Ophthalmologic examination found no abnormalities in the fundus or in intraocular pressure, while visual field and visual acuity tests were not completed because of non-cooperation. Brain MRI showed marked diffuse cortical and subcortical atrophy in bilateral parietal and occipital lobes. [^18^F]FDG PET revealed significant hypometabolism in diffuse regions of bilateral temporal, parietal, and occipital lobes, prominently in occipital lobes, and no metabolic changes were observed in frontal lobes, basal ganglia, and thalami; [^18^F]PI2620 PET showed increased tracer uptake throughout the brain, especially in temporal, parietal, and occipital lobes, while standardized uptake value ratios (SUVRs) were 3.8, 4.0, and 4.3, respectively, using cerebellum as the reference region, in close correspondence with the regions that showed the most prominent hypometabolism; [^18^F]AV45 PET was positive with amyloid deposited throughout the cortices (Figure 1). Whole-exome sequencing identified the p.Pro354Leu (c.1061C>T) variant of the microtubule-associated protein tau (*MAPT*) gene. The apolipoprotein E genotype was ε3/ε4. No other variant was detected in genes associated with early-onset AD [amyloid precursor protein (*APP*), presenilin 1 (*PSEN1*), and *PSEN2*]; frontotemporal dementia and parkinsonism linked to chromosome 17 [granulin (*GRN*), fused in sarcoma (*FUS*), TAR DNA-binding protein (*TARDBP*), charged multivesicular body protein 2b (*CHMP2B*), valosin-containing protein (*VCP*), etc.]; and other degenerative diseases. There was no expansion detected in Chromosome 9 open reading frame 72 [*C9ORF72*]. Genetic testing and neurologic evaluation were also proposed to the patient's parents, but they refused consent.

The patient was diagnosed with PCA and received treatment with donepezil 10 mg/day and memantine 10 mg/day; additionally, psychoeducation and home-based cognitive retraining tasks were assigned to the patient and his family members, but the symptoms deteriorated.

The clinical study protocol and informed consent forms were approved by the ethics committee of Xuanwu Hospital of Capital Medical University, China. Written informed consent was obtained from the patient and his legal guardian for [^18^F] PI2620 PET, genetic testing, and the publication of this case report (including all data and images).

## Discussion

In this report, we described a case characterized by predominant visuoperceptual and visuospatial deficits that fulfilled the diagnostic criteria for PCA (1). And the beta amyloid deposition, pathologic tau, and neurodegeneration (A+/T+/N+) fulfilled the 2018 National Institute on Aging and Alzheimer's Association (NIA-AA) guidelines, which confirmed that, in this patient, PCA was attributed to AD (8). Furthermore, the tau load revealed by [^18^F]PI2620 PET corresponded to prominent visual cognitive impairment, the extent of regional brain atrophy, and regions of hypometabolism in the [^18^F]FDG-PET scan.

In our patient, early diagnosis was confounded by the detection of p.P354L variant of *MAPT*, while subsequent [^18^F]AV45 PET confirmed the AD pathology. Mutations in *MAPT*—which encodes tau protein—are considered as a pathogenic factor in frontotemporal dementia (FTD) but have seldom been observed in PCA (9). Only one PCA patient carrying an *MAPT* p.V363I mutation has been reported (10). However, the variant detected in our patient (p.P354L) has not been previously reported and has uncertain significance according to the variant interpretation guidelines of the American College of Medical Genetics and Genomics (11). So, it was not clear whether FTD tau pathology existed as a comorbidity in this patient.

Amyloid aggregation and tau hyperphosphorylation are typical pathophysiologic events in PCA with AD pathology. In recent years, various tau imaging markers have become available to investigate tau pathology. The most extensively used ligand is [^18^F]AV1451. Tau PET studies in PCA using [^18^F]AV1451 have indicated that the tracer is taken up and selectively retained in bilateral parietal, occipital, and occipitotemporal cortices, corresponding to the regions that are clinically affected and consistent with the patterns of functional and structural deterioration and tau deposition detected in postmortem studies. However, measurable off-target binding to MAO-A or MAO-B, neuromelanin- and melanin-containing cells, choroid plexus, basal ganglia, and some other areas without tau pathology was also detected by [^18^F]AV1451 PET, affecting the quantitative measurement of tau burden, especially in target regions (5, 12, 13). In this study, we used [^18^F]PI2620, a novel tau tracer, to detect tau deposition *in vivo*. *In vitro* and animal studies have shown that [^18^F]PI2620 is highly selective for pathologic tau aggregates with no significant off-target binding to β-amyloid, MAO-A, or MAO-B and can be rapidly washed out from unaffected brain regions (5, 14). In-human clinical studies confirmed that tracer uptake level generally corresponds to disease severity throughout the course of AD and can clearly distinguish AD patients from healthy controls (6, 7). One study of four PCA cases showed robust increases in [^18^F]PI2620 uptake throughout the posterior cortical regions with relative sparing of the medial frontal and temporal lobes, which was related to disease severity (6); this is the only study to date assessing tau burden in PCA by [^18^F]PI2620 PET. In accordance with previous studies, in our patient, [^18^F]PI2620 uptake was more restricted to bilateral occipital cortices, consistent with the observed visuospatial and visuoperceptual deficits and brain atrophy and hypometabolism detected by MRI and [^18^F]FDG PET, respectively. Due to being in the later stage of disease, apart from prominent visual impairment, the patient also exhibited memory impairment and executive dysfunction, which was in line with brain atrophy, hypometabolism, and [^18^F]PI2620 uptake in bilateral temporal and parietal cortices.

This is the first study evaluating tau burden *in vivo* using a novel tau tracer, [^18^F]PI2620, in a Chinese PCA patient in relation to detailed clinical manifestations, neuropsychologic test results, and MRI, [^18^F]FDG PET, and [^18^F]AV45 PET findings. Our results demonstrate a clinical anatomical correspondence between the neuropsychological deficits, the tau uptake, and signs of neurodegeneration such as atrophy and hypometabolism seen in the MRI and [^18^F]FDG PET. Detection of tau aggregation has important implications for understanding the pathologic mechanisms underlying the development of PCA, as well as for disease monitoring and targeted therapy. A limitation of our study is that it involved a single case, and the patient was in the later phase of disease, which may suggest that the results must be validated by a larger sample study with varied clinical stages; nonetheless, it supports the clinical use of [^18^F]PI2620 for evaluating tau aggregations in living patients with PCA.

## Data Availability Statement

The original contributions presented in the study are included in the article/supplementary materials, further inquiries can be directed to the corresponding author/s.

## Ethics Statement

The studies involving human participants were reviewed and approved by Xuanwu Hospital of Capital Medical University, China. The patients/participants provided their written informed consent to participate in this study. Written informed consent was obtained from the individual(s) for the publication of any potentially identifiable images or data included in this article.

## Author Contributions

YK contributed to the patient review and medical review of the manuscript. LW and YK contributed to the study supervision and coordination. KX, XL, and YW contributed to genetic testing, mutation sequencing, and manuscript writing. HQ, YC, and DJ contributed to analysis and interpretation of brain MRI data. All authors read and approved the manuscript.

## Conflict of Interest

The authors declare that the research was conducted in the absence of any commercial or financial relationships that could be construed as a potential conflict of interest.
